# Reliability of Wearable-Sensor-Derived Measures of Physical Activity in Wheelchair-Dependent Spinal Cord Injured Patients

**DOI:** 10.3389/fneur.2018.01039

**Published:** 2018-12-10

**Authors:** Sophie Schneider, Werner L. Popp, Michael Brogioli, Urs Albisser, László Demkó, Isabelle Debecker, Inge-Marie Velstra, Roger Gassert, Armin Curt

**Affiliations:** ^1^Spinal Cord Injury Center, Balgrist University Hospital, Zurich, Switzerland; ^2^Rehabilitation Engineering Laboratory, Department of Health Sciences and Technology, ETH Zurich, Zurich, Switzerland; ^3^REHAB Basel, Clinic for Neurorehabilitation and Paraplegiology, Basel, Switzerland; ^4^Clinical Trial Unit, Swiss Paraplegic Center, Nottwil, Switzerland

**Keywords:** spinal cord injury, rehabilitation, physical activity, intervention studies, wearable sensors, reliability

## Abstract

Physical activity (PA) has been shown to have a positive influence on functional recovery in patients after a spinal cord injury (SCI). Hence, it can act as a confounder in clinical intervention studies. Wearable sensors are used to quantify PA in various neurological conditions. However, there is a lack of knowledge about the inter-day reliability of PA measures. The objective of this study was to investigate the single-day reliability of various PA measures in patients with a SCI and to propose recommendations on how many days of PA measurements are required to obtain reliable results. For this, PA of 63 wheelchair-dependent patients with a SCI were measured using wearable sensors. Patients of all age ranges (49.3 ± 16.6 years) and levels of injury (from C1 to L2, ASIA A-D) were included for this study and assessed at three to four different time periods during inpatient rehabilitation (2 weeks, 1 month, 3 months, and if applicable 6 months after injury) and after in-patient rehabilitation in their home-environment (at least 6 months after injury). The metrics of interest were total activity counts, PA intensity levels, metrics of wheeling quantity and metrics of movement quality. Activity counts showed consistently high single-day reliabilities, while measures of PA intensity levels considerably varied depending on the rehabilitation progress. Single-day reliabilities of metrics of movement quantity decreased with rehabilitation progress, while metrics of movement quality increased. To achieve a mean reliability of 0.8, we found that three continuous recording days are required for out-patients, and 2 days for in-patients. Furthermore, the results show similar weekday and weekend wheeling activity for in- and out-patients. To our knowledge, this is the first study to investigate the reliability of an extended set of sensor-based measures of PA in both acute and chronic wheelchair-dependent SCI patients. The results provide recommendations for sensor-based assessments of PA in clinical SCI studies.

## Introduction

Neurological disorders such as Spinal Cord Injury (SCI) are characterized by the different degrees of impairment of motor and sensory function. Earlier studies have investigated the impact of physical activity (PA) on functional recovery and found a positive effect in various neurological diseases (1–3). Past intervention studies in SCI focused on the integration of activity-based therapies with various intensities, duration, and type of PA, into rehabilitation programs to improve functional recovery. The outcome of these studies, however, are contradictory, with some of them showing improved strength or functional ability of the upper limbs (4–7) and performance in daily life (8), whereas others could not show any significant effect on the functional recovery (9, 10). One reason for such divergent results could be the subjective and non-comprehensive assessments of PA performed by the patient outside the controlled interventions. Thus, PA needs to be objectively assessed to better estimate the effects of interventions and the impact of PA on patient recovery in general.

In the past 15 years, accelerometers and inertial measurement units (IMUs) have been introduced to quantify PA more objectively. The use of accelerometers is well established in health sciences, especially in quantifying PA in the able-bodied population (11), elderly (12), children (13), and patients with various neurological conditions such as stroke (14, 15), Parkinson's (16), and multiple sclerosis (17). In SCI, studies have been conducted to develop metrics to capture PA in wheelchair-bound SCI patients (18, 19).

The levels of PA change throughout the rehabilitation process due to neurological recovery and compensation (20, 21), and can differ between individuals (22). Furthermore, they may vary from day to day as well due to environmental factors, but also due to patient characteristics like motivation, or general health status and pain. Therefore, there is a need to quantify how much the PA varies between single days within one patient and how many days are required to account for this variability to obtain a reliable representation of the overall PA level of the subject.

Guidelines on how many days the PA has to be monitored to obtain a reliable representation of the overall PA already exist for healthy adults and children. For healthy adults, a measurement period of 1 week has been suggested (23), while a measurement period of up to 11 days has been suggest for children (24). In older adults, a desired measurement duration of one to 2 days has been reported to achieve good reliabilities for sedentary, low and moderate-to-vigorous physical activity (25). In neurological diseases, e.g., in multiple sclerosis, guidelines suggest 4–6 days for sedentary behavior and 3–7 days for low and moderate-to-vigorous physical activity (26). The existing guidelines, however, cannot easily be translated to the SCI population, and especially not to wheelchair-dependent patients because of the completely different PA patterns such as wheeling instead of walking.

Because of the novelty of PA research in SCI, no comprehensive guidelines on measurement periods exist for this population. Sonenblum et al. (27) proposed a measurement period of 1 week to obtain reliable estimations of PA related solely to wheelchair usage, such as distance wheeled and duration of wheeling episodes. Yet, this conclusion is drawn from a limited number of patients with different neurological conditions, and only in their chronic stages. Since the variability between days might change between the stages and it might also be different for the different metrics of PA, we propose guidelines for the wheelchair-dependent patients on how many days of measurement are required for various measures of PA during the different stages of rehabilitation after the incidence of SCI. The primary aim of this study was to estimate the reliability of several sensor-based metrics of PA at different time points during the rehabilitation progress. The secondary aim was to compare the reliability of PA measures across days of active rehabilitation and on weekends.

## Methods

### Patients

In total, 63 patients with SCI were included in this analysis, participating in two observational studies (for information about the protocol see Measurement procedure).

Patients suffering from a traumatic or non-traumatic acute SCI with all NLI and levels of lesion completeness were admitted to this study. Any neurological disease other than SCI, and any orthopedic or psychiatric disorders, were considered as exclusion criteria. Additionally, only wheelchair-dependent patients, defined by a value of < 3 in all the mobility domains (12, 13, and 14) of the Spinal Cord Independence Measure III (SCIM III) (28) were considered for the analysis.

The NLI and completeness of the lesion (AIS) was assessed following the International Standards for Neurological Classification of SCI (ISNCSCI) (29). Patients with an NLI from C1 to Th1 were classified as tetraplegic, while patients with an NLI from Th2 to S2 were classified as paraplegic. Recruitment took place from 2014 until 2017, at the sites of the Swiss Paraplegic Center in Nottwil, the Rehab Basel in Basel and the Balgrist University Hospital in Zurich, Switzerland. All patients signed a written consent before participating in the study in accordance with the Declaration of Helsinki. The study was approved by the ethical committees of the cantons of Zurich (KEK-ZH-Nr. 2013-0202), Lucerne (EK 13018), and Basel (EK 34313) and is registered on clinicaltrials.gov (Identifier: NCT02098122).

### Measurement Procedure

For this study, the ReSense modules (30) were used as a measurement device. The ReSense modules are compact IMUs recording 3D acceleration, 3D angular velocity, 3D magnetic field strength, and barometric pressure for more than 24 h continuously. By turning all sensors except the accelerometer off, the battery life can be extended to over 2 weeks. In this study, only the acceleration data were used.

**Figure 1:**
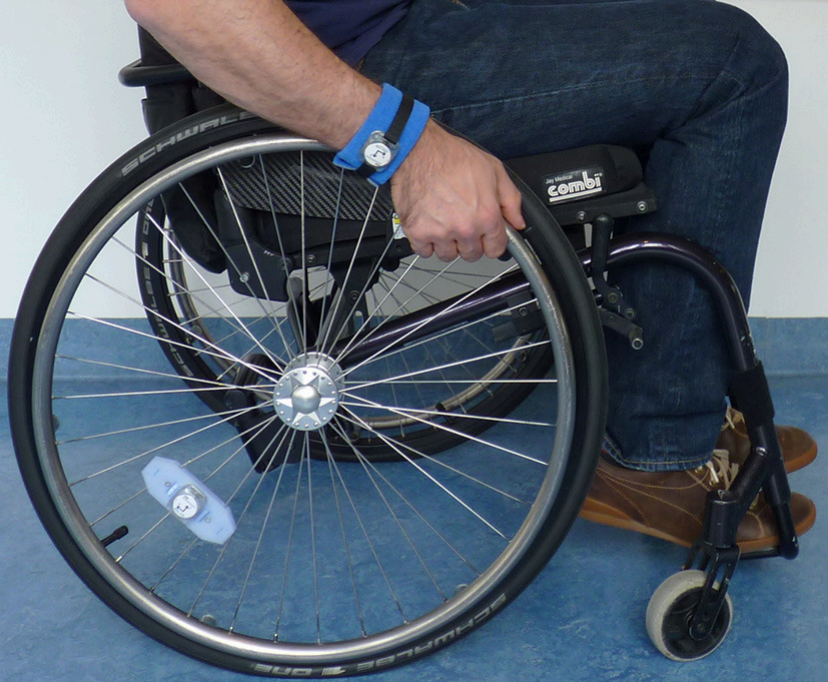
Photograph of one examiner wearing the sensors. One sensor was attached to the right wheel of each wheelchair, one sensor was attached to each wrist.

At all time points, patients were equipped with several ReSense modules (Figure 1). One sensor measuring acceleration was attached to each wrist with AlphaStrap Blue (North Coast) and Velcro Straps (Velcro) for a duration of three consecutive weekdays to capture upper limb movements. Patients were asked to wear the sensors continuously for about 72 h during day- and nighttime and just take them off for showering or swimming activities. They were told that their amount of activity was being measured and that they should engage in their everyday life actives. Due to the limited battery lifetime, the sensors were exchanged once a day and recharged. Additionally, one module measuring acceleration was mounted on the right wheel of each wheelchair for the duration of seven consecutive days to capture wheeling metrics precisely (18, 31).

**Figure 2:**
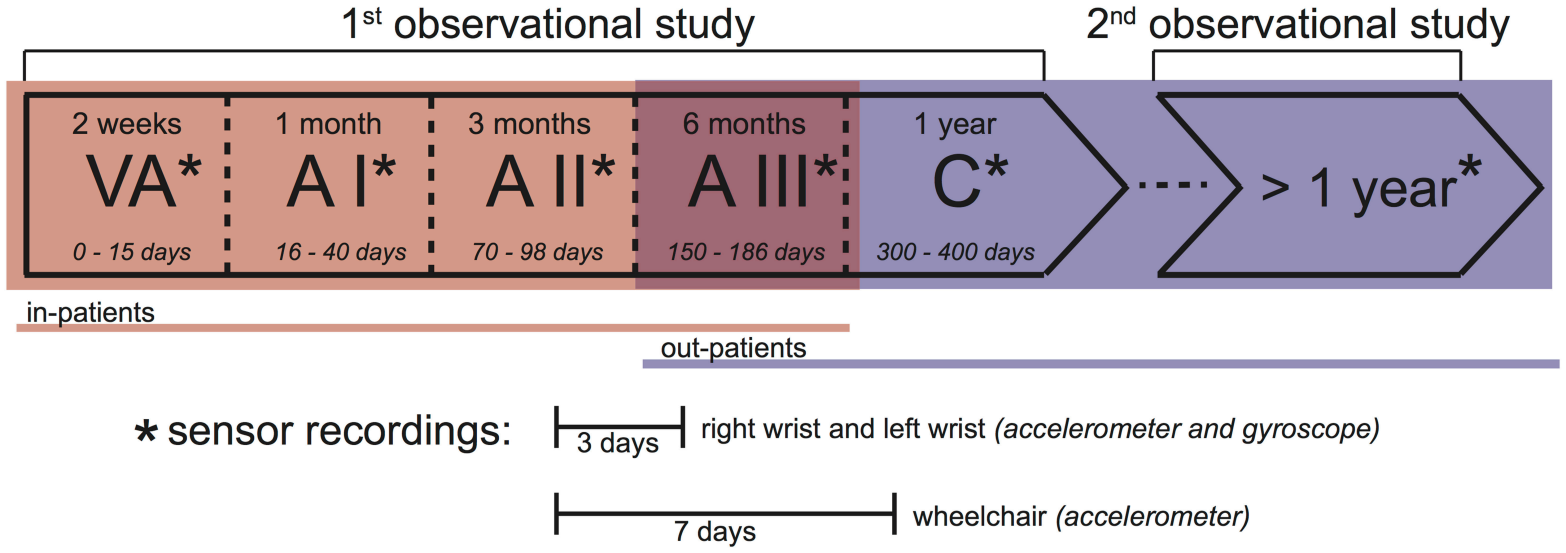
Measurement protocol. This study consists of two observational studies. In the 1st observational study, patients were measured at 5 time points during the rehabilitation process. In the 2nd observational study, a different patient cohort was measured only once, at least 1 year after injury. In stages VA, A I, A II, and partly A III of the 1st observational study, patients were in-patients (red). In the 2nd observational study, as well as partly in A III, and stage C of the 1st observational study, patients were out-patients (blue). At each time point (^*^), acceleration and angular velocity of the right and left wrists were recorded for 3 days, while the acceleration of the right wheel of the wheelchair was recorded for 7 days. Overall upper limb activity (AC) and PA based on energy expenditure (SED, LPA, and MVPA) were calculated based on the 3 day recordings. All wheeling-related measures (DIST_TOT_, DIST_ACT_, and VEL) were calculated based on the 7 day recordings.

Data collection was conducted in the context of two observational studies (Figure 2). In the first observational study, patients were measured at five different time points during rehabilitation, each time for 3 consecutive days wearing the wrist sensors, and 7 consecutive days using the wheelchair sensor, respectively. The first four time points were within the clinical rehabilitation facilities (“in-patient”), whereas the last time point took place after discharge (“out-patient”). The in-patient rehabilitation was divided into four distinct stages, which conform to the time windows of the European Multicenter Study about SCI (EMSCI^1^:): very acute (VA), acute 1 (A I), acute 2 (A II), and acute 3 (A III), which are 2 weeks (0–15 days), 1 month (16–40 days), 3 months (70–98 days), and 6 months (150–186 days after injury), respectively. The last time point (out-patient) was defined to be 1 year after injury (chronic stage–C, 300–400 days). It is important to note that at stage A III, some patients were already discharged from the rehabilitation facility and were therefore analyzed within the out-patient group. Dividing the rehabilitation process into different stages has the advantage of stratifying the clinical picture of the patients and enables to analyze the patient data in short time windows in which a minimal functional change can be expected. In the second observational study, different patients were measured once after discharge for 3 consecutive days wearing the wrist sensors, and 7 consecutive days using the wheelchair sensor, respectively, at least 1 year after injury.

Not every patient was measured at every timepoint due to later recruitment or early drop-out and the number of patients of the 3-day measurement can differ from the number of the patients of the 7-day measurement due to technical issues with the sensors (Table 1).

**Table 1:** Patient demographics for all patients included (third row) as well as split up into patients included included in the 1st and 2nd observational studies for 3-day as well as 7-day measurements (bold values).

				**Females**		**Tetraplegics**		**Age [years]**	
		**N**		**N (%)**		**N (%)**		**Mean** **±** **sd**	
**Total patients**		**63**		**17 (27)**		**34 (54)**		**49.3** **±** **16.6**	
		**3-day**	**7-day**	**3-day**	**7-day**	**3-day**	**7-day**	**3-day**	**7-day**
**1st observational study**		**41**	**42**	**12 (29)**	**12 (29)**	**26 (63)**	**27 (64)**	**49.4** **±** **19.4**	**49.5** **±** **19.2**
Very acute (VA)		10	10	2 (20)	2 (20)	7 (70)	7 (70)	43.0 ± 16.5	43.0 ±16.5
Acute 1 (A I)		36	36	11 (31)	11 (31)	22 (61)	23 (64)	48.0 ± 19.4	48.2 ± 19.0
Acute 2 (A II)		21	24	7 (33)	8 (33)	12 (57)	14 (58)	53.2 ± 18.0	52.1 ± 18.5
Acute 3 (A III)	Total	17	20	6 (35)	7 (35)	10 (59)	11 (55)	49.1 ± 19.8	48.1 ± 19.4
	In	14	16	4 (29)	4 (25)	10 (71)	11 (69)	50.1 ± 18.3	48.8 ± 18.8
	Out	3	4	2 (67)	3 (75)	0 (0)	0 (0)	44.0 ± 30.3	45.3 ± 24.9
Chronic (C)		5	4	1 (20)	0 (0)	3 (60)	3 (75)	42.6 ± 18.0	40.8 ± 20.3
**2nd observational study**		**22**	**19**	**5 (23)**	**5 (26)**	**8 (36)**	**8 (42)**	**49.5** **±** **11.5**	**51** **±** **11.3**
In-patient^*^		81	86	24 (30)	25 (29)	51 (63)	55 (64)	49.1 ± 18.5	48.8 ± 18.4
Out-patient^*^		30	27	8 (27)	8 (30)	11 (37)	11 (41)	47.8 ± 14.5	48.7 ± 14.9

* pooled data set.

*Detailed numbers are given for all single stages constituting the 1st observational study: 2 weeks after injury (VA), 4 weeks after injury (A I), 3 months after injury (A II), 6 months after injury (A III), and 1 year after injury (C). In stage A III, in- as well as out-patients are included. For a combined analysis of all in-patients, data of stages VA, A I, A II, and A III (partly) were pooled. Data of stages A III (partly), stage C, as well as the 2nd observational study were pooled for a combined analysis of all out-patients. “Tetraplegics” are defined by a lesion level from C1 to Th1*.

### Data Analysis and Statistics

The number of included patients varied depending on the specific analysis and time point (Table 1). For the analyses focusing on the whole in-patient group, data from stages VA, A I, A II, and partly A III of the 1st observational study were pooled. Similarly, data of the whole out-patient group were pooled from the 2nd observational study, and from stage A III (partly) and C of the 1st observational study.

#### Preprocessing

The desired sampling rate of the sensor was 50 Hz. However, the exact sampling rate of the ReSense sensors can vary between 49 and 51 Hz. Therefore, the raw data was resampled to 50 Hz using a common set of time points for all the modules that were used together (32). The periods of not wearing the sensors were removed from the data using a semi-automatic algorithm. The algorithm labels periods with 20 min of consecutive zero-counts as potential non-wear times (33). Thereafter, the labeled periods were visually inspected by an expert and manually adapted where necessary.

#### Sensor-Based Metrics

Sensor-based metrics were divided into 4 major categories: activity counts of overall upper limb movement, PA intensity levels (time spent in sedentary PA, low PA and moderate-to-vigorous PA), metrics of wheeling quantity (total and actively wheeled distance), and metrics of movement quality (upper limb movement laterality and mean wheeling velocity as a proximate of wheeling performance).

##### Overall upper limb activity

Activity counts (***AC***) were used to enumerate total forearm activity in a generalized way and were calculated by applying the discrete integral over the acceleration magnitude in epochs with the length of 1 min (34) and subsequently averaging the AC values over all epochs. ***AC*** of the right and left wrist were summed up.

##### PA intensity levels

Different intensity levels of PA were defined by using AC cut-off values. These cut-off values were derived from previous energy expenditure measures in combination with IMU data (35). The intensity levels were defined by means of the metabolic equivalence of task (MET) adapted for SCI (36), where sedentary activities (***SED***) corresponded to a MET level below 1.5, low physical activity (***LPA***) to a MET value between 1.5 and 3, and moderate-to-vigorous activities (***MVPA***) corresponded to a MET level above 3. SED, LPA, and MVPA are expressed in minutes spent in the respective intensity level per 24 h.

##### Metrics of wheeling quantity

To calculate wheeling-related metrics, a previously published algorithm (18) was used to (i) detect the phases of wheeling activity by applying heuristic rules, and (ii) to classify these phases into active and passive wheeling by using support vector machine classifiers. The total distance (**DIST**_TOT_) and the distance wheeled actively (**DIST**_ACT_) were extracted from the data and normalized to 24 h.

##### Metrics of movement quality

Whereas, the three aforementioned categories described how often movements were performed, the following metrics describe how the movements were performed.

Upper limb movement laterality (***LAT***) represents the symmetry of upper limb movements in general. LAT was calculated by computing the AC in epochs of 2 s for the right and left hand, dividing AC of the right hand and left hand and log transforming this ratio. The median value of the absolute log transform was used for the analysis. Details about the calculation can be found in Brogioli et al. (19). Scores for ***LAT*** range from minus to plus infinity quantifying the amount of ***LAT***, with zero for no ***LAT***.

Mean velocity (***VEL***) can be interpreted as a proximate measure for the quality of wheeling. Patients with improved functional ability will be able to wheel on average faster than patients in earlier stages of rehabilitation, or with more severe impairments.

***VEL*** was defined as the mean absolute velocity of active propulsion, and was extracted using the aforementioned wheeling algorithm (18).

#### Statistics

First, the single-day reliabilities of all sensor-based metrics were calculated. Then the number of days needed for a reliable measurement was identified.

Single-day reliabilities for ***AC***, ***SED***, ***LPA***, ***MVPA***, and ***LAT*** were calculated based on the 3-day measurements, because they require information of the wrist sensors. Single-day reliabilities for ***DIST***_***TOT***_, ***DIST***_***ACT***_, and ***VEL*** were calculated based on the 7-day measurements, because they require information of the wheel sensor only.

Single-day reliability was defined as the Intraclass Correlation Coefficient (ICC), which was calculated using a variance portioning approach based on a one-way random effects model, with the random effect being on the subject level (37)

(1) $$\begin{array}{l} \text{ICC}=\frac{\sigma_{\text{s}}^{2}}{\sigma_{\text{s}}^{2}+\sigma_{\text{res}}^{2}}, \end{array}$$

where $\sigma_{\text{s}}^{2}$ is the between-subject variance and $\sigma_{\text{res}}^{2}\;$the residual variance. This approach is a well-established method especially in the field of PA research (23, 38, 39).

The confidence intervals for ICC were calculated based on the exact confidence limit equation (40).

According to Koo and Li (41), ICC values higher than 0.9 are considered as excellent, between 0.75 and 0.9 as good, between 0.5 and 0.75 as moderate, and lower than 0.5 as poor reliability.

To calculate the number of days needed for a reliable measurement (N), the Spearman Brown prophecy formula was used (42),

(2) $$\begin{array}{l} \text{N}=\;\frac{{\text{ICC}}_{\text{t}}\cdot \left(1-{\text{ICC}}_{\text{s}}\right)}{{\text{ICC}}_{\text{s}}\cdot \left(1-{\text{ICC}}_{\text{t}}\right)}, \end{array}$$

where ICC_t_ is the desired level of reliability and ICC_s_ is the single-day reliability. The desired reliability was set to 0.8, which is considered as an acceptable value according to literature (43).

To assess the relation of the wheeling-related metrics during weekdays and the weekend, equivalence tests were used. For normally distributed data, the Two Sided *T*-test (TOST) approach was used (44). In TOST, an epsilon (ε) has to be defined that corresponds to the level of practical equivalence (LOPE). We chose ε as the mean value of all the standard deviations of the respective metric:

(3) $$\begin{array}{l} \varepsilon =\frac{\sum_{i=1}^{n}\sigma_{i}^{metric}}{n}, \end{array}$$

where *n* is the number of patients, and $\sigma_{i}^{metric}\;$is the standard deviation of the *i*-th subject for the metric of interest.

For non-normally distributed data, the TOST procedure was adapted by using the non-parametric Mann-Whitney-Wilcoxon Test instead of the Student's *t*-test.

A sample size calculation was performed according to the method presented in (45). The results of this analysis can be found in Table S1.

Preprocessing and calculation of the output metrics was conducted using MATLAB R2017a (MathWorks, Natick, MA, USA). Statistics were computed using R (The R project for Statistical Computing, R Core Team).

## Results

### Patient Characteristics

The mean age of all patients was 49.3 ± 16.6 years at the time of recruitment. 17 (27%) of the patients were female. ASIA impairment scale (AIS) levels ranged from A to D, (A: 27, B: 9, C: 16, and D: 11 patients at the time of recruitment) and the neurological level of injury (NLI) from C1 to L2 (C1–C4: 17, C5–C8: 17, T1–T5: 6, T6–T12: 19, and L1–L2: 4 patients at the time of recruitment). More detailed information about patient numbers and demographics can be found in Table 1.

### Single-Day Reliabilities

**Figure 3:**
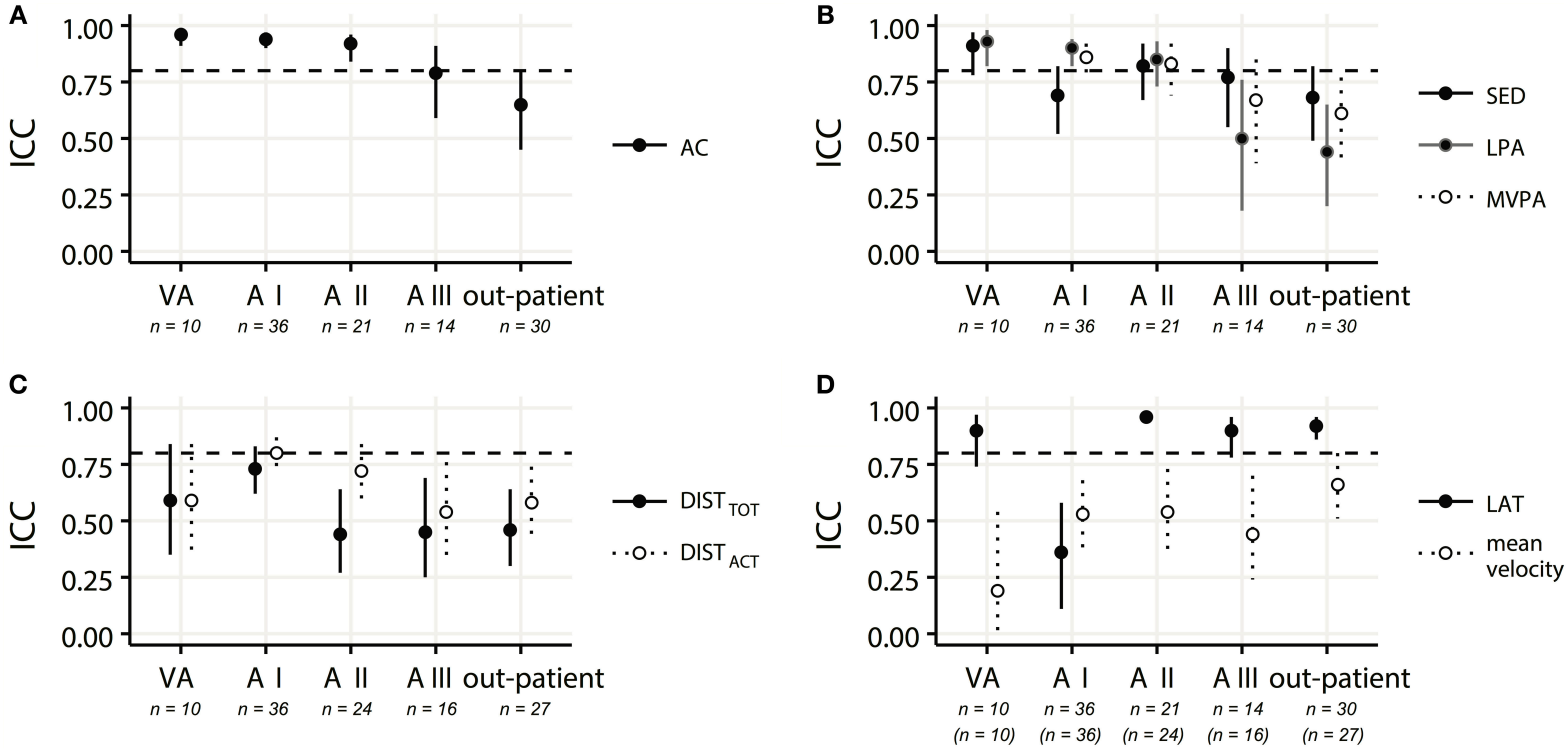
ICC values representing the single-day reliabilities for **(A)** activity counts (AC); **(B)** time spent in sedentary activity (SED), low physical activity (LPA), and moderate-to-vigorous activity (MVPA); **(C)** total distance traveled in a wheelchair (DIST_TOT_) and distance traveled actively in a wheelchair (DIST_ACT_); and **(D)** laterality (LAT) and mean velocity (VEL) for all in-patient rehabilitation stages (very acute (VA−2 weeks after injury), acute I (A I−4 weeks after injury), acute II (A II−3 months after injury), acute III (A III−6 months after injury), as well as for the out-patients (> 6 months after injury). The horizontal dashed lines depict the ICC level of 0.8, which was chosen as a requirement for a reliable measurement. Solid and dotted lines indicate the confidence intervals. Indicated patient numbers n are the pooled numbers.

Single-day reliabilities of metrics of PA varied depending on the time after SCI (i.e., rehabilitation progress) ranging from excellent to poor reliability levels (Figure 3). ICC of metrics describing movement quantity (***AC***, ***SED***, ***LPA***, ***MVPA***, ***DIST***_***TOT***_, and ***DIST***_***ACT***_) tended to decrease during the rehabilitation progress (Figures 3A–C) and decreased e.g., from excellent reliability levels (0.93) for ***LPA*** in stage VA to poor levels (0.44) for ***LPA*** in out-patients. In contrast, measures describing movement quality (***LAT*** and ***VEL***) tended to increase during rehabilitation (Figure 3D). Especially, reliability of ***VEL*** improved from a poor level of the ICC (0.19) at stage VA to a moderate level (0.66) for out-patients. Overall upper limb activity (AC) showed excellent ICC levels (ICC > 0.92) during the first three acute stages with a decrease at later stages of rehabilitation to a good level (0.79) and a moderate level (0.65) after discharge (out-patient).

**Figure 4:**
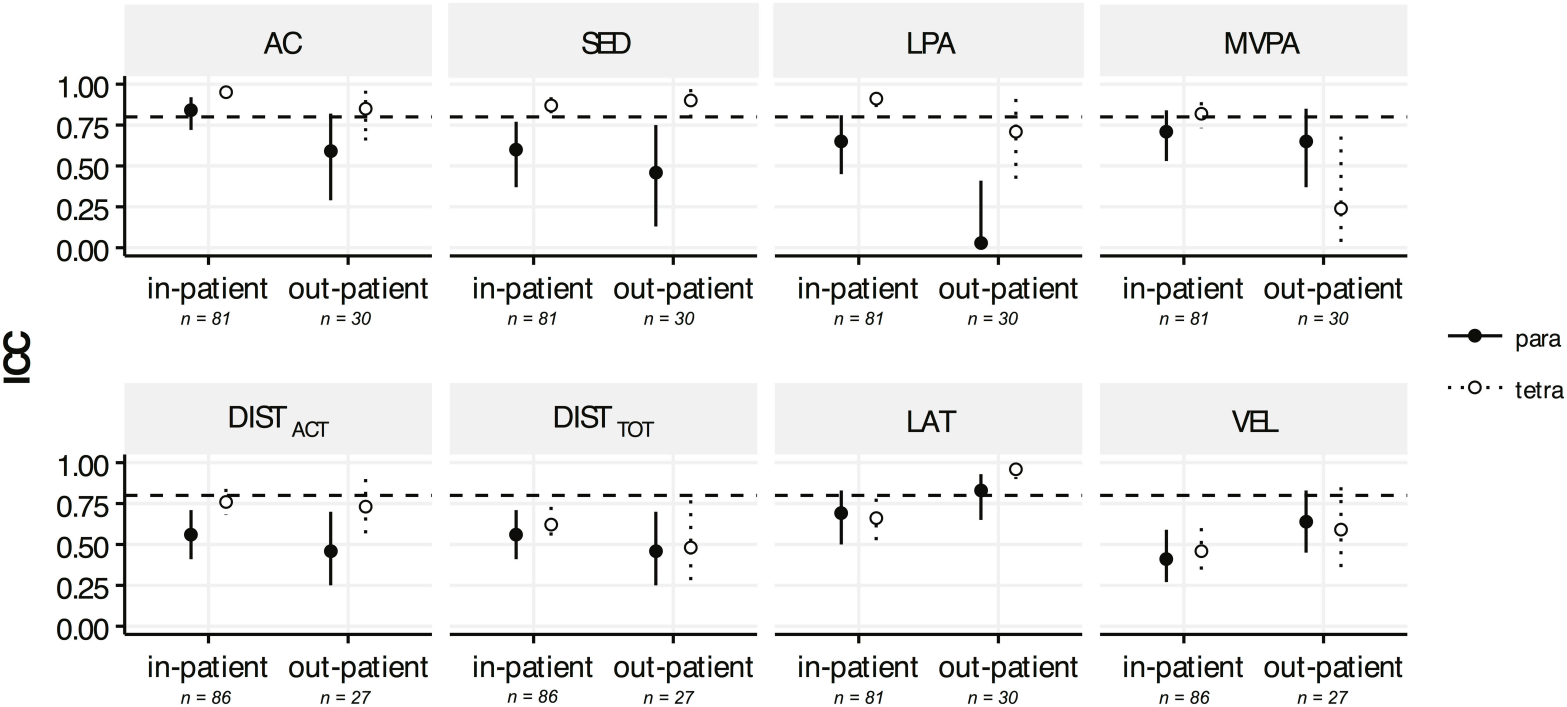
ICC values representing the single-day reliabilities for activity counts (AC), time spent in sedentary activity (SED), low physical activity (LPA), moderate-to-vigorous activity (MVPA), total distance traveled in a wheelchair (DIST_TOT_), distance traveled actively in a wheelchair (DIST_ACT_), laterality (LAT), and mean velocity during active wheeling (VEL) for wheelchair-dependent paraplegic patients (full circle, solid lines) compared to wheelchair-dependent tetraplegic patients (empty circle, dotted lines) for the in-patients (from 2 weeks after injury to 6 months after injury) and out-patients (> 6 months after injury). The dashed horizontal lines depict the ICC level of 0.8, which was chosen as a requirement for a reliable measurement. Solid and dotted lines indicate the confidence intervals. Indicated patient numbers n are the pooled numbers.

Overall single-day reliabilities were higher in tetraplegic patients than in paraplegic patients for most metrics (Figure 4). One exception to this was found in the reliability of MVPA in the out-patients, were the single-day reliability for tetraplegic patients was poor (0.24) and thus lower than the moderate level (0.65) for the paraplegic patients. Furthermore, the single-day reliability of LPA is poor (0.03) in paraplegic out-patients.”

### Required Number of Days

**Figure 5:**
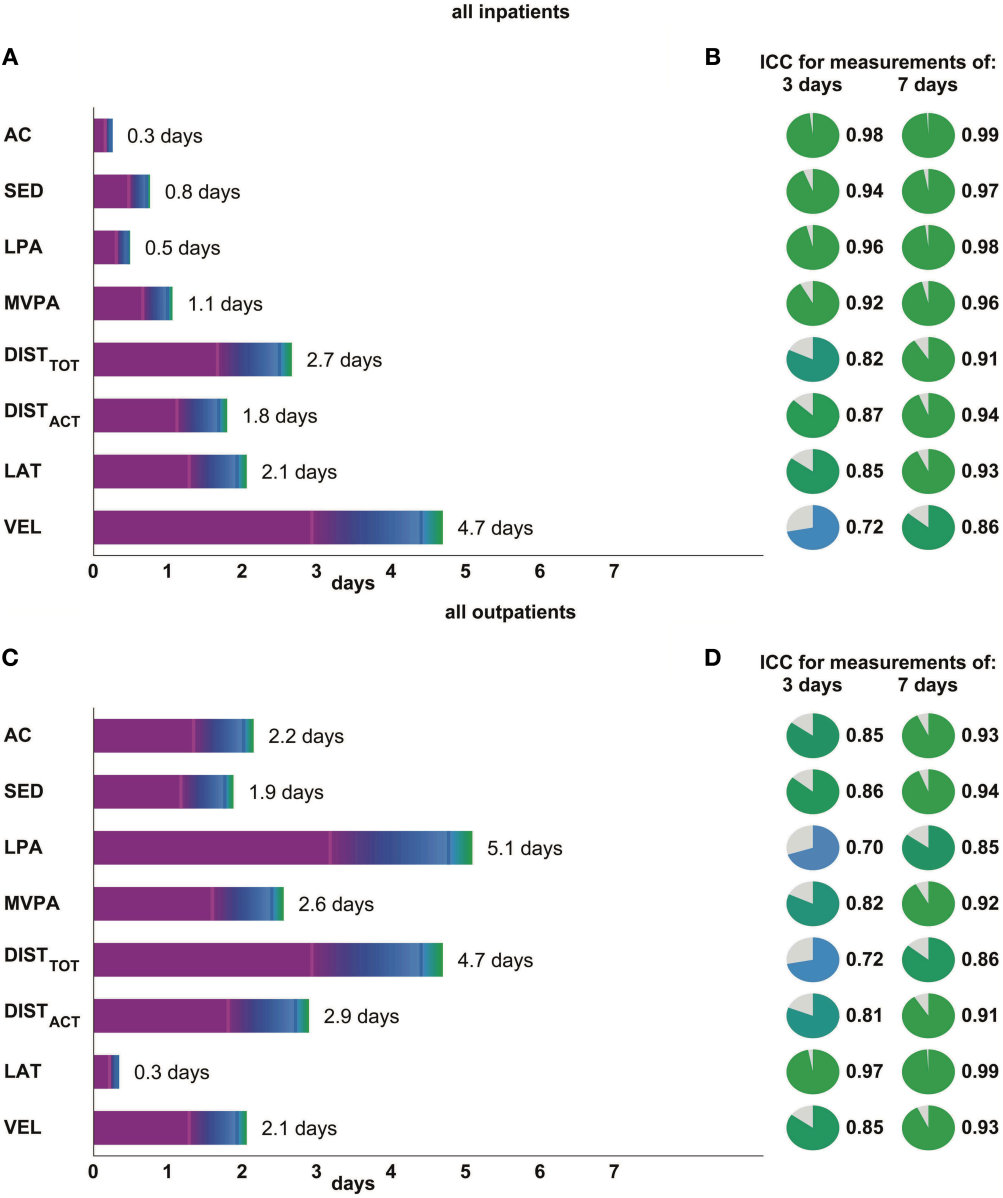
The subfigures on the left side (**A**: in-patients, **C**: out-patients) represent the number of measurement days needed in order to achieve a reliability of 0.8 for different metrics of movement quantity (activity counts – AC, time spent in sedentary activity – SED, in low physical activity – LPA, in moderate-to-vigorous activity – MVPA, total distance wheeled – DIST_TOT_, and distance wheeled actively – DIST_ACT_) as well as metrics of movement quality (laterality – LAT and mean wheeling velocity – VEL). Additionally, the numbers of measurement days needed for a reliability of 0.5 and 0.75 are presented with magenta and blue vertical bars, respectively. The subfigures on the right side (**B**: in-patients, **D**: out-patients) show the reliabilities, which would be achieved when measuring 3 and 7 days, respectively.

A mean reliability of 0.8 is reached when monitoring in-patients for 2 days and out-patients for 3 days for all metrics (Figures 5A,C). A 7-day measurement is estimated to reach excellent reliabilities for all metrics in both in- and out-patients (Figures 5B,D).

### Influence of Weekday vs. Weekend

**Figure 6:**
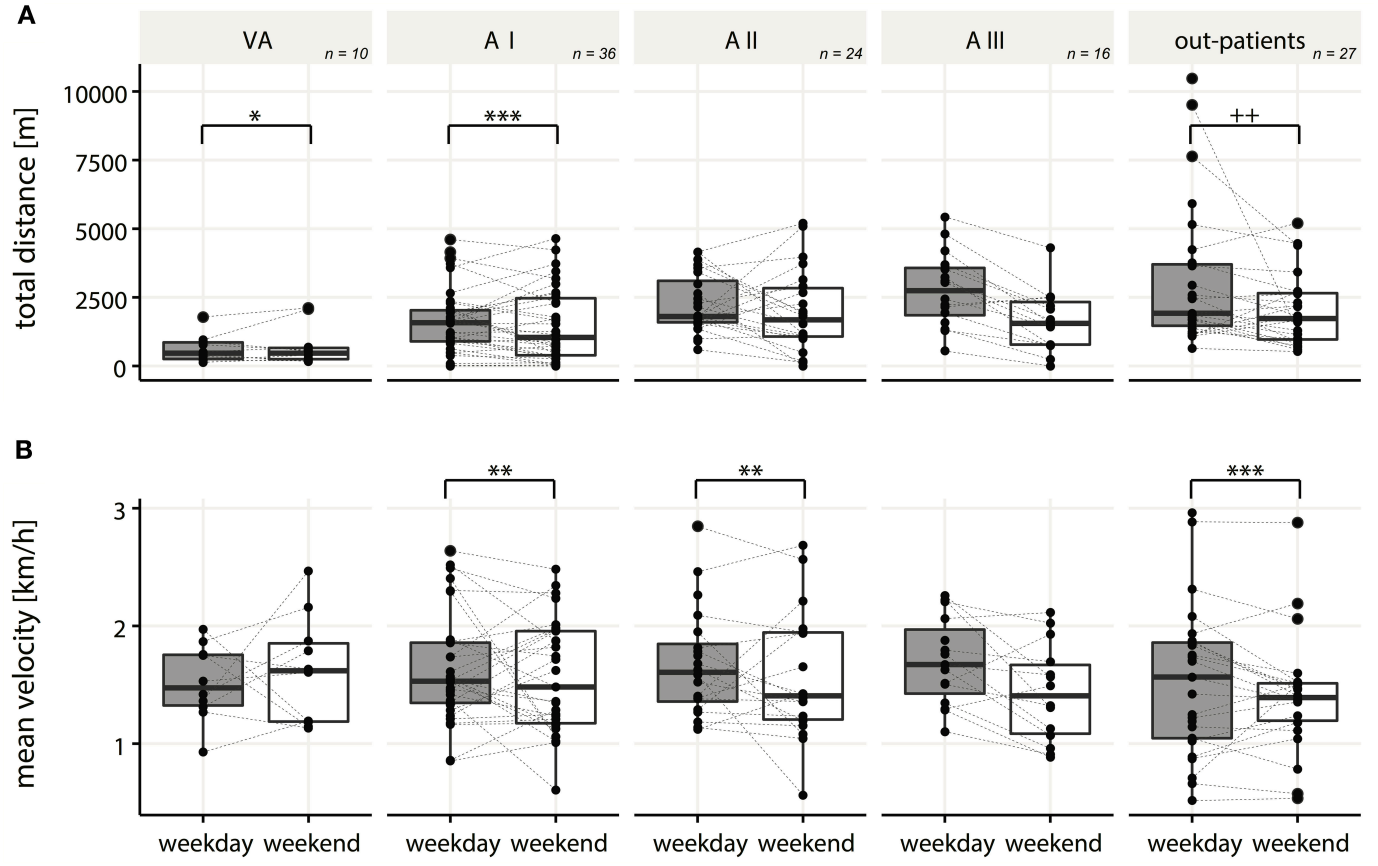
Boxplots for total distance traveled in a wheelchair **(A)** and mean velocity during active wheeling **(B)** during weekdays vs. weekends in all single in-patient stages (VA, AI, AII, AIII), as well as out-patients. ^*^/^+^ denotes p-value of < 0.05, ^**^/^++^ a *p*-value of < 0.01, ^***^/^+++^ a *p*-value of < 0.001, respectively. *P*-values were calculated using the TOST procedure for normally distributed data (^*^), respectively, the adapted equivalence test based on the Mann-Whitney-Wilcoxon Test for non-normally distributed data (^+^).

With the chosen LOPEs and a significance level of 0.05, equivalence could be established for ***DIST***_***TOT***_ as well as ***DIST***_***ACT***_ between weekdays and the weekend in all in-patients and out-patients, as well as single stages VA and A I (Table 2, Figure 6A). At stages A II and A III, no equivalence could be shown (Figure 6A) for ***DIST***_***TOT***_ and ***DIST***_***ACT***_. Results for active distance are very similar to total distance for all stages, and thus not presented.

**Table 2:** Descriptive statistics for total distance traveled in a wheelchair (**DIST**_TOT_), distance traveled actively in a wheelchair (**DIST**_ACT_), and mean velocity during active wheeling (**VEL**) performed during weekdays and weekends (mean ± SD and median (IQR) for pooled in-patients and out-patients, as well as all single stages, VA (very acute−2 weeks after injury), A I (acute I−4 weeks after injury), A II (acute II−3 months after injury), A III (acute III−6 months after injury).

	**Workday mean (± SD)**	**Weekend mean (± SD)**	**Workday median (IQR)**	**Weekend median (IQR)**	**Limit of practical equivalence (± LOPE)**	**Confidence Interval**	**Equivalence *p*-value**
**DIST**_TOT_ **[m]**							
In-patients	1910.9 ± 1271.6	1562.3 ± 1312.9	1722.1 (1631.64)	1184.1 (1828.9)	± 750.1	127.8, 538.7	0.001
VA	625.7 ± 499.8	723.2 ± 740.7	467.1 (605.3)	467.3 (409.1)	± 368.6	−338.9, 143.8	0.035
A I	1644.3 ± 1170	1501.1 ± 1321	1576.2 (1133.9)	1040.3 (2078.3)	± 615.1	−148.3, 315.1	<0.001
A II	2264.5 ± 1059.6	1980.9 ± 1502.9	1803.3 (1512.3)	1681.3 (1763.7)	± 744.6	−251.9, 875.6	0.1
A III	2783.7 ± 1346.8	1646.5 ± 1095.4	2742.5 (1724.1)	1551.9 (1550.7)	± 1277.5	817.2, 1618.6	0.399
Out-patients	3365.2 ± 2698.3	2139.9 ± 1367	2462.9 (1367)	1812.4 (621.9)	± 1092.3	31.5, 772.9	0.003
**DIST**_**ACT**_ **[m]**							
In-patients	1686.4 ± 1396	1379 ± 1270.9	1446.8 (1927.7)	1100.2 (1841.7)	± 661.2	130.6, 464.7	<0.001
VA	625.3 ± 499.5	722.9 ± 740.6	467.1 (604.9)	466.8 (408.9)	± 368.6	−338.9, 143.7	0.035
A I	1494.9 ± 1268.1	1370.2 ± 1370.7	1225.8 (1495)	819.8 (2202.4)	± 537.4	−151.8, 264.2	<0.001
A II	1836.8 ± 1427.7	1508.9 ± 1375.6	1690.6 (2562.7)	1160.2 (1466)	± 610.2	−77.6, 712.2	0.108
A III	2555.2 ± 1528.4	1645.7 ± 1094.9	2329.7 (2250)	1551.5 (1546.9)	± 1147	700.9, 1458.9	0.38
Out-patients	2738.7 ± 2605.2	1871.1 ± 1561.7	2443 (2565.7)	1727.8 (1946)	± 672.2	−16.6, 550.3	0.012
**VEL [km/h]**							
In-patients	1.64 ± 0.43	1.54 ± 0.48	1.54 (0.52)	1.43 (0.77)	± 0.36	0, 0.19	<0.001
VA	1.52 ± 0.32	1.62 ± 0.46	1.47 (0.43)	1.62 (0.66)	± 0.41	−0.42, 0.22	0.055
A I	1.63 ± 0.47	1.56 ± 0.48	1.53 (0.51)	1.48 (0.78)	± 0.35	−0.06, 0.25	0.004
A II	1.68 ± 0.46	1.57 ± 0.55	1.61 (0.49)	1.41 (0.74)	± 0.36	−0.11, 0.26	0.009
A III	1.7 ± 0.37	1.43 ± 0.41	1.67 (0.54)	1.41 (0.58)	± 0.36	0.09, 0.43	0.161
Out-patients	1.53 ± 0.64	1.41 ± 0.51	1.57 (0.81)	1.39 (0.32)	± 0.32	−0.06, 0.17	<0.001

*The last three columns contain the calculated limits of practical equivalence (LOPE) used for the equivalence tests, the confidence intervals, and the p-values resulting from the equivalence tests. ^a^denotes p-values resulting from the Mann-Whitney-Wilcoxon Test of equivalence for non-normally distributed data, the remaining p-values were calculated using the TOST procedure for normally distributed data*.

For ***VEL***, equivalence could be shown in all in-patients and out-patients, as well as at single stages A I, A II, whereas in stage VA and A III no equivalence could be established (Table 2 and Figure 6B).

## Discussion

To our knowledge, this study is the first to investigate the reliabilities of a comprehensive set of sensor-based measures of PA in both acute and chronic wheelchair-dependent SCI patients. These findings provide recommendations for the application of sensor-based assessments of PA that enable non-obstructive long term recordings throughout clinical studies in in- and out patients.

### Single-Day Reliabilities

Single-day reliabilities of PA metrics depend highly on the clinical condition, i.e., the stage of rehabilitation and the extent of functional impairment. The reliability of measures of movement quantity such as activity counts of upper limb activity, PA intensity levels and wheeling-related metrics decreased during in-patient rehabilitation and in the out-patient setting, while in general was found to be higher for tetraplegic patients. One reason for this might be that in-patients have a more regular daily schedule due to preplanned therapy sessions as observed by therapists at the different rehabilitation centers, resulting in lower variability of daily activities. Reliabilities of metrics of movement quantity are higher in patients with a higher impairment like in tetraplegia and in the early stages of rehabilitation, as the use of the upper limbs for these patients is mostly limited to the very structured therapy sessions, lowering the variability between single days. Additionally, these patients might also reach their upper limits of PA during their daily schedules, resulting in a very low variability between single days.

High reliability levels of upper limb activity in terms of ***AC*** compared to the other quantitative metrics suggest that this measure, although widely used in PA-research, may provide a rather rough approximation of PA levels in SCI patients, lacking the detailed information about PA intensity patterns and specific movements like wheeling.

Metrics based on PA intensity levels show lower single-day reliability levels than ***AC***, suggesting that these metrics capture more detailed information about PA levels which likely vary between single days. Another possible explanation could be that the AC thresholding for this analysis introduces noise into the estimates. Future studies are needed to investigate this in more detail.

The reliability of ***LPA*** in paraplegic out-patients was considerably lower compared to the remaining metrics based on PA intensity levels. Since the reliability is calculated by dividing the between-subject variance by the total variance (Equation 1), for ***LPA*** in paraplegic out-patients, the poor reliability can be explained by a very low variance between the individual patients compared to the variance between the single days. While tetraplegic patients show a much higher between-subject variance, this might be a hint that ***LPA*** could be influenced by the level of impairment of the upper limbs. ***LPA*** is likely to represent activities of daily living, as demonstrated earlier (35). Assuming that a large amount of activities of daily living (not involving mobility), e.g., feeding, showering, and dressing are equally presented in each patient, ***LPA*** should not vary strongly between single patients. This hypothesis is valid for patients that are not impaired in the upper-limbs, i.e., paraplegics, as can be seen in the low variability of ***LPA*** between the patients (***LPA***: SD: 1.1h, Min: 9h, Max: 13h). In contrast, the level of impairment of the upper limbs varies strongly in the tetraplegic group, which might be the reason for a higher variability of ***LPA*** between the patients (***LPA***: SD: 2.8h, Min: 6h, Max: 15 h).

In contrast to the increased reliability in ***LPA*** for tetraplegic outpatients, ***MVPA*** shows a decreased single-day reliability in these patients. A possible reason is that these patients are challenged to even reach moderate-to-vigorous intensities (46, 47) and thus show it only occasionally and not in everyday PA.

In contrast to metrics of movement quantity, single-day reliabilities of metrics of movement quality like ***LAT*** and ***VEL*** increased during the rehabilitation and stayed on a higher level after discharge. During in-patient rehabilitation, patients learn various skills to handle their impairment, e.g., wheeling techniques or compensatory strategies for activities of daily living, which may result in higher variability between single days at the earlier stages of rehabilitation. Moreover, at the beginning of the rehabilitation process arm rehabilitative training is often unilateral (e.g., with ArmeoPower (48) training), leading to a high discrepancy of ***LAT*** between specific therapy sessions and leisure time and thus increasing the variability of ***LAT*** between different days. Furthermore, the therapy schedules may vary strongly between different days at these stages, which can result in more variable measures of movement quality on different days due to dedicated rehabilitation sessions with specific training aims such as improving the function of the more impaired side in tetraplegics leading to a higher variability in measures of movement quality as can be seen for ***LAT*** in the AI stage. After the patients learn certain strategies, they may apply them more consistently during their daily activities, resulting in higher single-day reliabilities at the later stages of rehabilitation. The high reliability of ***LAT*** in the stage VA might be due to the fact that patients are mainly bound to the bed, and not showing much PA in general, which may lead to a higher reliability.

### Required Number of Days For Reliable Measures

Based on our data, metrics of movement quality should optimally be measured for 4 days to achieve a mean reliability of 0.8, which is commonly used in the field of PA research (23, 24, 26, 49). In the in-patient setting, we suggest measuring metrics of movement quantity for 2 days to achieve a mean reliability of 0.8, while in the out-patient setting measuring on average for 3 days is required to achieve the same reliability. The findings of high reliability of 2-days recordings increase the applicability of sensor measurements in the clinical routine where, especially in acute patients, wearing sensors for too long may be an additional burden. However, we suggest 4 days to capture all analyzed metrics of movement quantity reliably, and one to 2 days for the measures of movement quality in the out-patient setting.

Measuring for 7 days would yield excellent reliabilities for all metrics in all patients, which might be relevant in research and clinical studies where even the detection of small changes in PA patterns may have an impact on outcomes. However, measurement duration is always in tradeoff with clinical applicability and patient compliance.

### Difference Between Weekdays And Weekend

We investigated whether it makes a difference to measure PA on weekends or during the week. For this, only wheeling-related metrics (***DIST***_***TOT***_, ***DIST***_***ACT***_, and ***VEL***) were analyzed, as 7-day recordings were only available from the wheel-mounted sensor. In in-patients as well as in out-patients we could show equivalence of ***DIST***_***TOT***_ and ***DIST***_***ACT***_ between weekdays and weekends. This suggests that measurements can be taken on any day of the week, keeping in mind that single days might represent unexpected outliers due to an event not occurring regularly. Nevertheless, the results for the in-patients have to be taken with precautions. Splitting up the in-patients into the single stages, equivalence between weekdays and weekends of ***DIST***_***TOT***_ and ***DIST***_***ACT***_ could only be shown at the very early stages of rehabilitation (VA and AI), suggesting that for the stages of A II and A III both weekdays and weekends should be measured in order to obtain a comprehensive picture of the patients' overall PA. During early phases of rehabilitation, patients typically receive individual therapy instead of group therapies and their therapy schedules are less tight as observed by therapist at the different centers. We hypothesize that this could explain the observation of similar amounts of activity on the weekends and during the week. At later stages, however, the therapy schedule of the patients gets tighter during the week, which is why they might use the weekends for recovery. The fact that some patients can leave the rehabilitation facility over the weekend at later stages of rehabilitation might have an additional impact on their different behaviors during weekdays and weekends.

One could hypothesize that in out-patients the wheeling distances differ during weekdays and weekends mainly due to the fact that patients might work during the week and thus show different activity patterns than on the weekends. However, in out-patients, equal wheeling distances (***DIST***_***ACT***_ and ***DIST***_***TOT***_) were found during the week and on the weekends, which might indicate that the patients we measured were not yet, or if then only partially back to work (50) or worked rather from home instead of having a working space away from home.

Equivalence of ***VEL*** could be shown in all in- and out-patients, suggesting that measurements can be taken on any day of the week to reliably capture ***VEL***. However, when examining individual stages of the in-patient rehabilitation, at stage VA as well as at A III, no equivalence could be shown. In the latter stage, patients showed a higher ***VEL*** during the week than on the weekends, which might be due to the integration of sports activities into their therapy schedule, as already shown for ambulatory SCI patients (51). The result found for the stage VA is based on only a limited number of observations as most of these patients do not wheel actively, and thus has to be taken with care.

### Comparison to Literature

Our results for the reliabilities of wheeling-related metrics in the out-patients are in line with literature, proposing up to 1 week of measuring wheeling-related PA in wheelchair-dependent chronic SCI patients (27). For ***AC*** as well as PA intensity times (***SED***, ***LPA***, and ***MVPA***), we can only compare our results to those of the able-bodied population. Single-day reliability for ***AC*** was found to be moderate in able-bodied individuals (23), which is consistent with our results in the out-patients. Similarly, single-day reliabilities for ***SED*** and ***MVPA*** are moderate and comparable to our results (23, 25). In contrast to a low single-day reliability found in our study, a good single-day reliability for ***LPA*** has been reported in the able-bodied population (23). This low single-day reliability happens to be distinctive to the wheelchair-dependent SCI population, and thus should not be compared to the able-bodied population.

To the best of our knowledge, this is the first work to analyze the reliability of physical activity metrics in the in-patient setting and thus no comparable data is available.

### Choice of Accelerometer Cut-Points

Cut-off points are commonly used to define intensity levels and were established in previous studies for the healthy population (52, 53) and for stroke survivors (54). However, appropriate cut-off values depend on populations and type of wearable sensors used (55). Furthermore, changes in those cut-off values directly influence the metrics of intensity levels (56). Therefore, we defined cut-off values specific for our population of interest and for the wearable sensor used in this study. We calculated our cut-off points based on indirect calorimetry values as commonly done in the field (52–56). Transferring our results to methodologies using different accelerometer cut-off points has to be done carefully, as the influence of the cut-off points on the reliability is unknown, and reliability values might change.

### Study Limitations

We would like to emphasize three main limitations of our study. The first one is the moderate sample size particularly in the very acute stage. Sample size is often a problem in SCI research. Especially in very acute stages recruitment of the patients and measuring these is challenging. In reliability studies, low sample size results in larger confidence intervals, making the interpretation of the results more difficult. Nevertheless, our sample size is reasonable if compared to other studies in the SCI population.

A further limitation is recording for only 3 days with the sensors attached to the wrists. Especially in tetraplegic patients, there is a risk of pressure sores caused by wearing the sensor straps for too long. Thus, a longer measurement time would expose the patients to an increased risk of damage to the skin. Furthermore, compliance decreases with increased number of measurement days. This limitation might result in larger confidence intervals.

A sample size calculation was conducted. Assuming an acceptable confidence interval width of 0.2, our sample sizes in the inpatient-setting are sufficient. In the outpatient setting, higher sample sizes would be required to make more precise statements. The problem of large confidence intervals in reliability studies has been addressed previously (45). This issue can be resolved by either increasing the number of subjects or the number of measurement days, and should be considered for further studies.

One limitation in studies using wearable sensors in general are possible behavioral reactions, i.e., subjects could alter their behavior because of the knowledge of being measured. Conflicting statements about the amount of reactivity have been made in literature (57, 58). However, the accuracy of PA measures based on wearable sensors is higher than the accuracy of the conventional questionnaires (59, 60) and thus better suitable to estimate PA levels.

Lastly, PA intensity levels were estimated from activity counts based on a previous study. Dedicated algorithms for the direct estimation of energy expenditure (35, 61) or direct measurements of energy expenditure might lead to slightly altered results, but the latter is very challenging to perform especially with acute patients due to the required equipment and the extensive protocol including standardized food intake and calibration phases.

## Conclusion

We conclude that single-day reliabilities of metrics to capture PA in acute and chronic wheelchair-dependent SCI patients vary considerably depending on the clinical setting. With increasing functional recovery of the patients, metrics of movement quantity tend to become less reliable, whereas metrics of movement quality become more reliable. Depending on the specific metrics, 2 days are required on average to capture PA reliably in in-patients, whereas 3 days are required for out-patients. Furthermore, we suggest using ***AC*** only as a rather general measure for assessing the overall PA level of patients, and only in combination with more detailed metrics, e.g., PA intensity levels and wheeling-related metrics. This avoids a possible loss of information about the variability of PA during a whole day. Our results are based on a reasonable sample size for this population and thus provide robust recommendations on how to design clinical studies investigating PA as a primary outcome, or as a confounder in intervention studies in order to better evaluate the actual intervention effect.

## Author Contributions

SS, WP, MB, RG, and AC designed the study. SS, WP, MB, UA, ID, and I-MV collected the experimental data. SS and WP analyzed the data. SS, WP, MB, UA, LD, ID, I-MV, RG, and AC interpreted the results. SS, WP, MB, UA, LD, ID, I-MV, RG, and AC revised the manuscript and approved the final version.

### Conflict of Interest Statement

The authors declare that the research was conducted in the absence of any commercial or financial relationships that could be construed as a potential conflict of interest.

## Footnotes

^1^www.emsci.org

## Abbreviations

SCI: Spinal Cord Injury
PA: physical activity
IMU: Inertial Measurement Unit
ICC: Intraclass Correlation Coefficient
***AC***: activity counts
***LAT***: laterality
MET: metabolic equivalent of task
***SED***: sedentary activity
***LPA***: low physical activity
***MVPA***: moderate-to-vigorous activity
***DIST***_***TOT***_: total distance
***DIST***_***ACT***_: actively wheeled distance
***VEL***: mean velocity during active wheeling.
