# Supplementary material for: Reliability of Wearable-Sensor-Derived Measures of Physical Activity in Wheelchair-Dependent Spinal Cord Injured Patients
Source: Front Neurol. 2018 Dec 10;9:1039. doi: 10.3389/fneur.2018.01039 (PMC6295582; doi:10.3389/fneur.2018.01039)
Supplement: Supplementary file 1 [file Table_1.DOCX]

**Table S1:** Sample size calculation for activity counts (AC), time spent in sedentary activity (SED), low physical activity (LPA), and moderate-to-vigorous activity (MVPA), total distance travelled in a wheelchair (DIST_TOT_), distance travelled actively in a wheelchair (DIST_ACT_), laterality (LAT) and mean velocity (VEL) for pooled in-and out-patients. The desired confidence interval width is set to 0.2.

|  | in-patients | out-patients |
| --- | --- | --- |
| AC | 6 | 91 |
| SED | 28 | 80 |
| LPA | 15 | 158 |
| MVPA | 42 | 105 |
| DIST_TOT_ | 66 | 81 |
| DIST_ACT_ | 49 | 69 |
| LAT | 89 | 9 |
| VEL | 82 | 56 |
